# Rapid Screening of Antimicrobial Synthetic Peptides

**DOI:** 10.1007/s10989-015-9494-4

**Published:** 2015-09-28

**Authors:** Maciej Jaskiewicz, Malgorzata Orlowska, Gabriela Olizarowicz, Dorian Migon, Daria Grzywacz, Wojciech Kamysz

**Affiliations:** Department of Inorganic Chemistry, Faculty of Pharmacy, Medical University of Gdansk, Al. Gen. J. Hallera 107, 80-416 Gdańsk, Poland; Lipopharm.pl, ul. Koscielna 16A, 83-210 Zblewo, Poland

**Keywords:** Antimicrobial peptides, Peptides, TLC, Direct bioautography, TLC-DB

## Abstract

Increasing resistance to conventional antibiotics among microorganisms is one of the leading problems of medicine nowadays. Antimicrobial peptides are compounds exhibiting both antibacterial and antifungal activities. However, it is difficult to predict whether a designed new compound would exhibit any biological activity. Moreover, purification of the peptides is one of the most time-consuming and expensive steps of the synthesis that sometimes leads to unnecessary loss of solvents and reagents. In our study we have developed a thin-layer chromatography (TLC) direct bioautography technique for rapid determination of antimicrobial activity of peptides without the necessity of high-performance liquid chromatography purification. In this assay, crude peptides were applied and separated on a TLC plate. Then, pre-prepared plates were dipped into microbial suspension and incubated under optimum conditions for bacteria and fungi as well. The activity of the tested compounds was visualized by spraying the TLC plates with a cell viability reagent, resazurin (7-hydroxy-3H-phenoxazin-3-one 10-oxide). Effectiveness of this assay was compared with minimal inhibitory concentration results obtained by broth microdilution assay. Interestingly, so far such a screening method has not been applied for this group of compounds.

## Introduction

Increasing resistance to antibiotics among microorganisms is one of the major problems in the course of effective therapy (Liu et al. 2014). Importance of this issue is still urgent, especially when considering the fact that antibiotics are widely available and frequently used (von Nussbaum et al. 2006). It is also related to an increasing number of immunocompromised patients (Helmehorst et al. Helmerhorst et al. 1999). Many research teams have been focused on the development of new antimicrobial drugs, which do not cause rapid resistance. Antimicrobial peptides (AMPs) are compounds with promising features in this area, suggesting that they would in the future replace traditional antibiotics.

Antimicrobial peptides can be found among all organisms including bacteria, plants and animals (Dawgul et al. 2014; Gottler and Ramamoorthy 2009; Izadpanah and Gallo 2005; Seto et al. 2007; Wade et al. 2000). Usually, they represent part of the organisms’ innate immune system. Predominantly, the synthesis of new peptides is based on compounds occurring in nature. Furthermore, they are being modified to provide enhanced antimicrobial activity and to improve stability. Their mode of action is commonly based on disruption of cell membrane, however it is different for each kind of peptide (Landreh et al. 2014). For example, LL-37 (human cathelicidin) causes membrane thinning (Dürr et al. 2006). In spite of some advantages, many AMPs are characterized by high toxicity against mammalian cells. This often eliminates their use as medicaments or restricts their application for external usage only. One of the lipopeptides, Polimyxin B, approved by FDA for the treatment of infections, is a compound primarily synthesized by *Bacillus polymyxa* strains (Cochrane and Vederas 2014). Since 1939, when AMPs named gramicidins were isolated from *Bacillus brevis*, the number of reported compounds among this group has increased over 2000 (Phoenix et al. 2013). Their promising antimicrobial properties indicated the need of extensive search for the new AMPs.

Notwithstanding, the amount of active compounds that can be extracted from natural sources (e.g. plants, microorganisms, gland secretions etc.) is too small and makes the process unprofitable. It often does not suffice to carry out microbiological tests, structural research or analytical tests. Therefore, specific methods of the synthesis have been developed and optimized enabling the production on a required scale. Peptide synthesis was developed at the beginning of the twentieth century. Nowadays, solution-based and solid-phase methods of the synthesis can be distinguished. The latter one, developed by Merrifield in the 1960s was a breakthrough providing elimination of undesirable reaction products. It is less time consuming and does not cause many processing problems, as in the case of solution-based synthesis. The procedure relies mainly on two techniques using Fmoc (fluorenylmethyloxycarbonyl) and Boc (*tert*-butyloxycarbonyl) protecting groups for side chain protection of amino acids. A detailed description of the methodology and protocols for the peptides synthesis by solid phase peptide synthesis (SPPS) can be found in the literature (Albericio 2004; Andersson et al. 2000; Galanis et al. 2009; Hojo et al. 2011).

Analysis and purification of the crude, synthetic peptides is carried out mostly by high performance liquid chromatography (HPLC). This technique is suitable for obtaining pure peptides, even on a large scale, owing to its high reproducibility, accuracy, speed of separation and detection. In the case of extremely small samples, the purification process by means of preparative chromatography is rather expensive and inefficient. In particular, this is important in synthesis of new peptides with the desirable properties, e.g. antimicrobial activity. Therefore, there is a necessity to develop methods that would enable separation of small amounts of obtained compound and carrying out the direct analysis (e.g. microbiological testing).

The development of thin-layer chromatography (TLC) in recent years, has led to coming back of this analytical method. An increasing access to new adsorption beds enables separation of different chemical structures, while maintaining selectivity and reproducibility. Introduction of high-performance thin-layer chromatography (HPTLC) has additionally improved the separation parameters. The advantages of TLC are: (1) possibility of carrying out the analysis of several compounds simultaneously, (2) reduction of eluent volumes, (3) retention of the process at any time, (4) ease of use, (5) wide range of available eluents and adsorbents. In addition, the main features distinguishing it among the chromatographic methods are: (1) less expensive eluents and apparatus, (2) samples do not need to be pre-purified, (3) determination of analyte before applying costly techniques such as HPLC or GC. TLC method has been successfully used for separation of many organic compounds such as vitamins, amino acids, peptides, alkaloids, flavonoids, carbohydrates, drugs etc. (Dolowy and Pyka 2014; Koch et al. 2013; Malinowska et al. 2013; Mohammad et al. 2012; Pyka 2014).

There are various methods that can be used for the detection of antimicrobial activity. We can distinguish dilution or agar diffusion techniques to determine minimum inhibitory concentration (MIC). Moreover, there are also bioautography methods allowing to detect trace amounts of antimicrobial compounds. For the first time, paper chromatography followed by bioautography was used by Goodall and Levi in 1946 to estimate the purity of penicillin. However, TLC plates for that purpose were used initially by Fisher and Launter in 1961 (Goodall and Levi 1946; Patil et al. 2013). Nowadays TLC bioautography techniques are commonly used for screening antimicrobial compounds derived from plant extracts or bacterial metabolites (Ebrahimipour et al. 2014; Guo et al. 2011; Kalaivani and Vidhya 2014; Patil et al. 2013). Among these techniques three approaches can be distinguished to identify antimicrobial activity of selected compounds namely contact bioautography (TLC-CB), immersion bioautography (TLC-IB) and direct bioautography (TLC-DB). TLC-DB enables both separation and microbial detection on a single TLC plate. Moreover, antimicrobial activity is visualized by growth inhibition zones of the tested microorganisms (Choma and Grzelak 2011).

In this study, TLC-DB method was employed using crude, synthetic, antimicrobial peptides that have recently been studied in our laboratory. To obtain more representative data, different TLC plates were taken under investigation as well as various classes of AMPs. So far, the bioautography assays have not been conducted for this group of compounds by any research team. Therefore it makes our study innovative. First of all, the purpose was preliminary determination of the activity of the peptides. This resulted in elimination of significant amounts of expensive reagents used for purification of crude peptides with doubtful antimicrobial activity. Moreover, using small amounts of crude compounds as well as solvents and reagents makes our method both economic and comparable with the green chemistry strategy.

## Materials and Methods

### Antimicrobial Peptides

All peptides: Citropin 1.1 (GLFDVIKKVASVIGGL-NH_2_), CAMEL (KWKLFKKIGAVLKVL-NH_2_), dimeric lipopeptide Laur-CKK-NH_2_ (Laur refers to lauric acid), lipopeptides: Pal-KK-NH_2_, Pal-K(TFA)K(TFA)-NH_2_, Pal-KGK-NH_2_ (Pal refers to palmitic acid and TFA refers to trifluoroacetic acid protecting groups on lysine), Mir-KGK-NH_2_ (Mir refers to myristic acid), ICHHCI-OH and p11 (QQRFEWEFEQQ-NH_2_) were synthesized by solid-phase method using Fmoc chemistry on a Polystyrene Amide AM-RAM resin (0.76 mmol/g), and for ICHHCI-OH on the 2-Cl-Trt-Resin. The synthesis consists of the following steps: (1) deprotection of the Fmoc group by 20 % (v/v) piperidine in DMF; (2) acylation with protected amino acid in a DMF/DCM solution with coupling agents: *N*,*N*′-diisopropylcarbodiimide (DIC) and 1-hydroxybenzotriazole (HOBt). Every step was preceded by rinsing the resin and running the chloranil test, respectively. Furthermore, the peptides were cleaved from the resin by using trifluoroacetic acid (TFA) and a mixture of scavengers: phenol, triisopropylsilane (TIS), water (92.5:2.5:2.5:2.5 v/v/v/v). Crude peptides were precipitated with cold diethyl ether and lyophilized. A selected part of the peptides was purified by reversed-phase high-performance liquid chromatography (RP-HPLC). The identity of all peptides was confirmed by mass spectrometry (ESI–MS).

### Antimicrobial Activity

Minimum inhibitory concentration (MIC) was determined by broth microdilution method on 96-well polypropylene plates, according to Clinical and Laboratory Standards Institute (CLSI) recommendations. All tests were conducted in triplicate and involved six reference strains of the following bacteria: *Bacillus subtilis* ATCC 6633, *Escherichia coli* ATCC 25922, *Klebsiella pneumoniae* ATCC 200603, *Pseudomonas aeruginosa* ATCC 9027, *Staphylococcus aureus* ATCC 25923, *Staphylococcus epidermidis* ATCC 14490 and two reference strains of fungi: *Candida albicans* ATCC 10231 and *Candida tropicalis* PCM 2681. Concentrations of applied peptides ranged from 512 to 1 µg/mL. Two microbiological media were used: Mueller–Hinton Broth for bacteria and Sabouraud Dextrose Broth for fungi. Incubation took 18 h at 37 °C and 48 h at 27 °C respectively. MIC was taken as the lowest concentration of a peptide where the visible growth of microbes was inhibited.

### Direct Bioautography

Direct bioautography assays to choose the most suitable and efficient stationary phase were performed on the following TLC plates: TLC Silica gel 60 RP-18 F254S (*Merck*), HPTLC-Platten Nano-SIL 20 UV_254_ (*Macherey*–*Nagel*), HPTLC Nano-ADAMANT UV_254_ (*Macherey*–*Nagel*) and TLC Aluminium oxide 60 F_254_ (*Merck*). The plates were sealed separately and autoclaved at 121 °C for 15 min. Peptide samples were dissolved in the solution of methanol and distilled water (75:25). 4 μL of each sample (5 mg/mL) was applied on the TLC plate. Separation was performed using a mobile phase consisting of *n*-butanol, acetone, acetic acid, 5 % ammonia and distilled water (4.5:1.5:1:1:2 v/v/v/v/v). Moreover, samples without separation were also investigated. In this case 10 µL of each sample was applied directly on the TLC plate and dried. All processes were performed under sterile conditions in the laminar flow cabinet. Microbiological assays were conducted using fivefold dilution of overnight culture of bacteria and a 48-h culture of fungi. After evaporation of eluents, TLC plates were sterilized under UV for 15 min and dipped into previously prepared inoculums of microorganisms for 20 s. Subsequently they were placed in a sterile Petri dish and incubated—bacteria at 37 °C for 5 h and fungi at 27 °C for 7 h. To visualize the growth inhibition zones, the plates were sprayed with 4 mg/mL (0.1 % Triton X-100) of a cell viability reagent, resazurin salt solution (*Sigma*-*Aldrich*).

## Results

### Antimicrobial Activity

All tested compounds have shown diverse antimicrobial activities. The most effective were CAMEL and lipopeptides Pal-KK-NH_2_, Pal-KGK-NH_2_ which inhibited growth of all bacterial strains and *C. albicans* at the lowest concentrations. ICHHCI, p11 and Pal-K(TFA)K(TFA)-NH_2_ were compounds with no antimicrobial activity among all the tested species of bacteria and fungi (Tables 1, 2) and they were used as negative control in bioautography assays.

**Table 1:** MIC values against reference strains of bacteria (µg/mL)

	*B. subtilis* ATCC 6633	*S. aureus* ATCC 25923	*S. epidermidis* ATCC 14490	*E. coli* ATCC 25922	*K. pneumoniae* ATCC 200603	*P. aeruginosa* ATCC 9027
CAMEL	2	4	1	2	2	256
Citropin 1.1	4	16	8	32	128	256
ICHHCI-OH	>512	>512	>512	>512	>512	>512
Laur-CKK-NH_2 dimer_	2	8	2	8	8	512
Pal-KK-NH_2_	2	8	4	8	16	128
Pal-K(TFA)K(TFA)-NH_2_	>512	>512	>512	>512	>512	>512
Pal-KGK-NH_2_	2	16	4	8	16	64
p11	>512	>512	>512	>512	>512	>512
Mir-KGK-NH_2_	2	16	4	64	128	>512

**Table 2:** MIC values against reference strains of fungi (µg/mL)

	*C. albicans* ATCC 10231	*C. tropicalis* PCM 2681
CAMEL	128	256
Citropin 1.1	128	128
ICHHCI-OH	>512	>512
Laur-CKK-NH_2 dimer_	512	>512
Pal-KK-NH_2_	128	128
Pal-K(TFA)K(TFA)-NH_2_	>512	>512
Pal-KGK-NH_2_	64	>512
p11	>512	>512
Mir-KGK-NH_2_	128	256

### Direct Bioautography

**Fig. 1:**
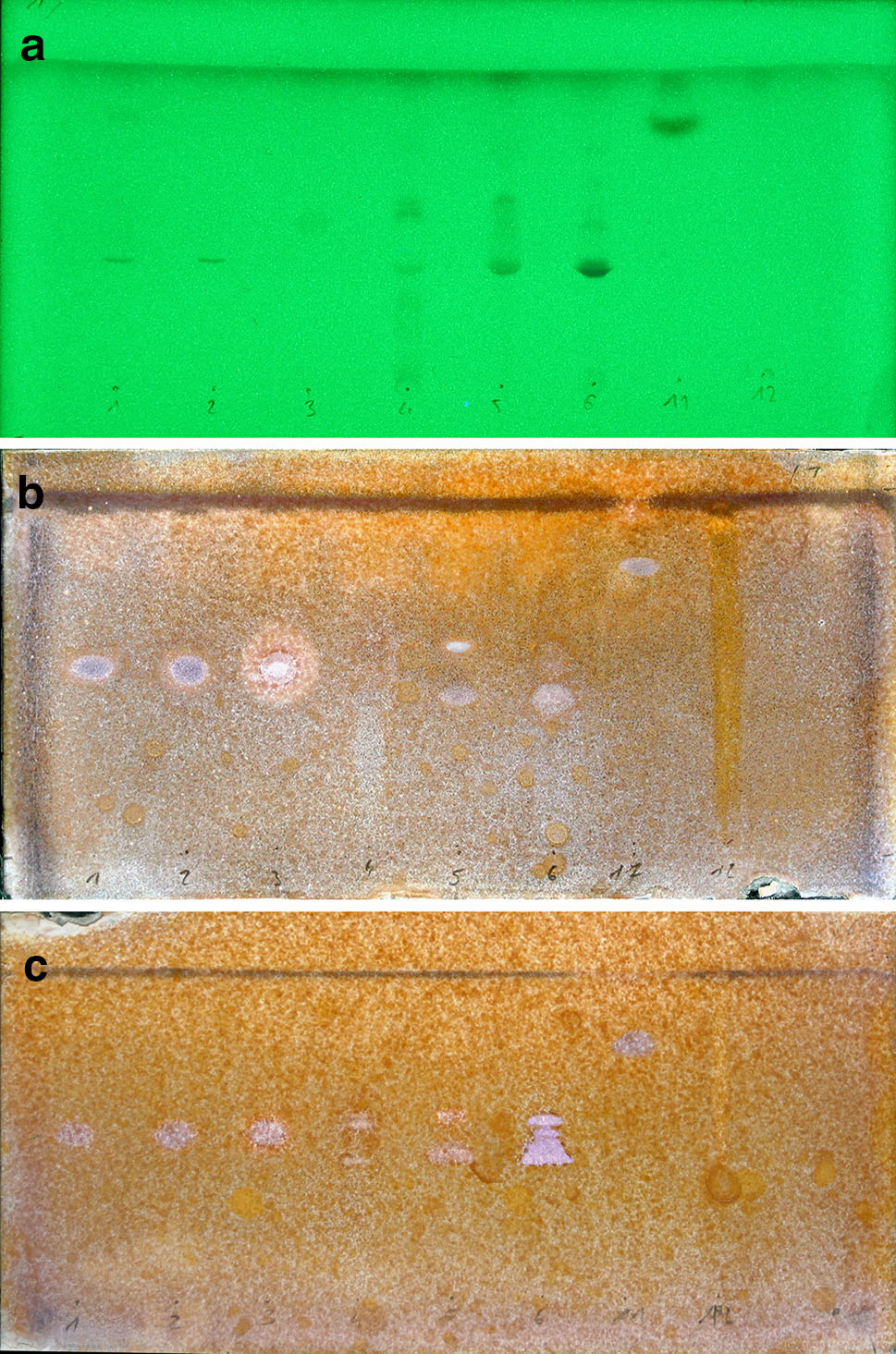
Plates with separation. 1—Pal-KGK-NH2; 2—Mir-KGK-NH2; 3—Citropin 1.1; 4—ICHHCI-OH; 5—Laur-CKK-NH2 dimer; 6—CAMEL; 11—Pal-KK-NH2; 12—Pal-K(Tfa)K(Tfa)-NH2. **a** Separation of peptides used in study (UV detection at 254 nm); **b** plate dipped in *S. aureus* ATCC 25923 inoculum after spraying with resazurin; **c** plate dipped in *C. albicans* ATCC 10231 inoculum after spraying with resazurin. Active compounds are visualized as *blue* growth-inhibition zones (Color figure online)

**Fig. 2:**
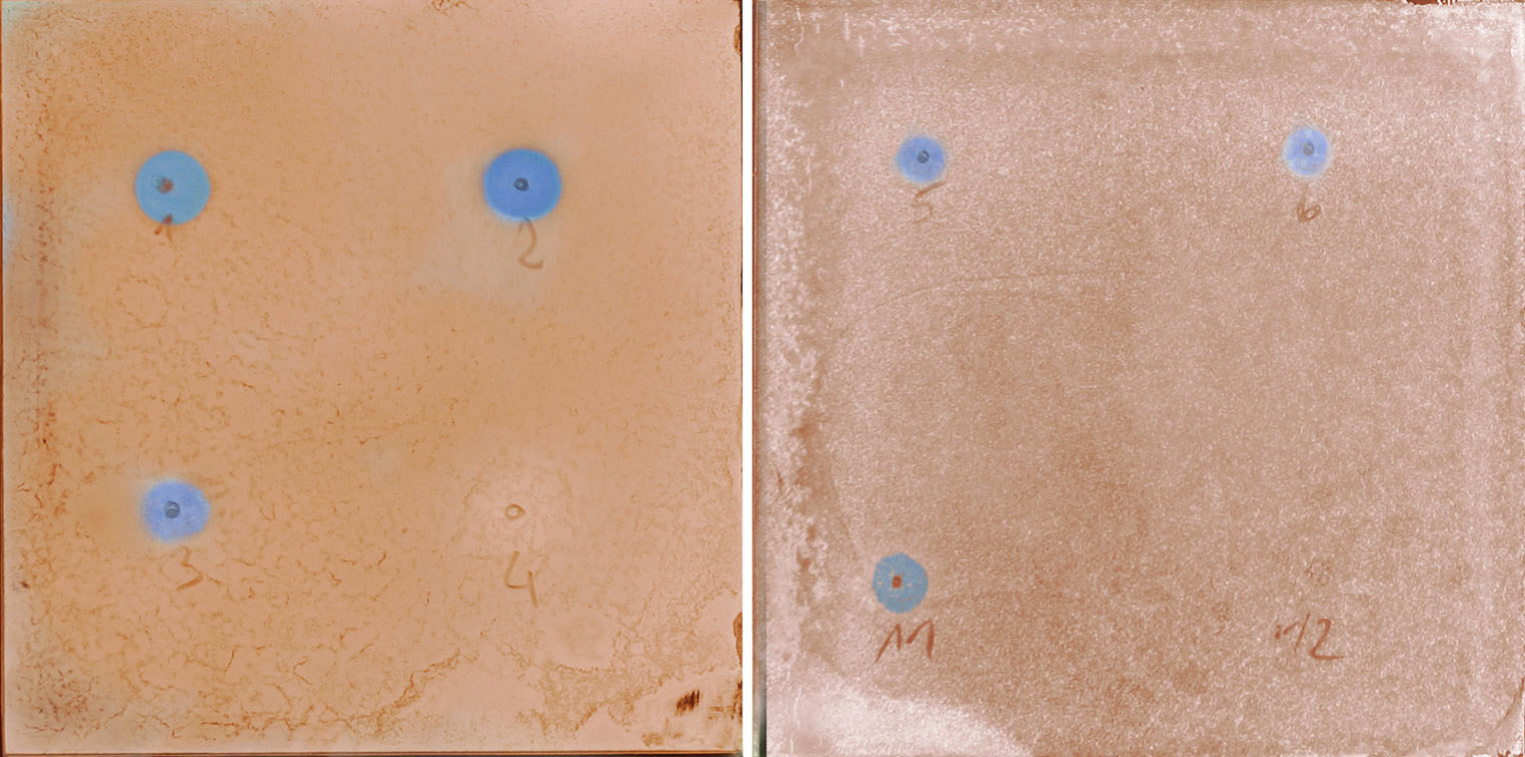
Plates without separation dipped in *E. coli* ATCC 25922 inoculum after spraying with resazurin. 1—Pal-KGK-NH2; 2—Mir-KGK-NH2; 3—Citropin 1.1; 4—ICHHCI-OH; 5—Laur-CKK-NH2 dimer; 6—CAMEL; 11—Pal-KK-NH2; 12—Pal-K(Tfa)K(Tfa)-NH2. Active compounds are visualized as *blue* growth-inhibition zones (Color figure online)

To determine the usefulness of the TLC layer material, preliminary separation experiments were carried out. Only Nano-SIL and Nano-ADAMANT layers have been found to be sufficiently effective. RP-18 layer’s stability was affected by TLC-bioautography procedures, resulting in material disruption. On the other hand, aluminum oxide layer was stable enough, however it gave low quality separation. Therefore, Nano-Sil and Nano-ADAMANT layers were chosen as the most suitable for TLC-DB of synthetic antimicrobial peptides. Mobile phase consisting of n-butanol, acetone, acetic acid, 5 % ammonia and distilled water (4.5:1.5:1:1:2 v/v/v/v/v) provided the best separation of tested compounds. Moreover, the influence of TFA content among crude peptides was also investigated. In this case all tested compounds were lyophilized from 0.1 mM HCl for three times before application on TLC plate in order to exchange the TFA to chloride counter ion. Nevertheless, the obtained results were the same. Assays with (Fig. 1) and without separation (Fig. 2) were performed simultaneously. Growing microorganisms that adhered to the surface of the TLC plate reduced the dye to pink resorufin. Spots where active compounds were applied or separated displayed blue, non-reduced growth-inhibition zones. The Rf values of non-viable cell area were the same for purified compounds. Interestingly, during development of the dimeric lipopeptide, Laur-CKK-NH_2_, two blue spots emerged one after the other. Moreover, during separation of the inactive peptide, ICHHCI-OH was spotted as a white reduction zone.

## Discussion

Direct bioautography techniques have been developed for antimicrobial activity screening of compounds occurring in nature such as plant extracts, essential oils or bacterial metabolites (Kalaivani and Vidhya 2014; Nostro et al. 2000; Patil et al. 2013; van Vuuren 2008). Synthetic antimicrobial peptides have often been designed on the basis of natural products. However, improvement of their activity involves many chemical modifications as they are often susceptible to inactivation under environmental conditions (e.g. pH, salinity, protease action) (Cruz et al. 2014; Lee et al. 1997; Werle and Bernkop-Schnurch 2006). So far, microbiological analysis of a large number of compounds is followed by massive loss of solvents, reagents, microbiological media and disposable laboratory materials. Moreover, results are obtained only after a specified time of incubation. There is a need for development of screening methods for the determination of antimicrobial activity in laboratories of peptide synthesis, especially with crude compounds, before their purification. TLC-DB appeared to be the most efficient and rapid method. In our study we have investigated the way which allowed to determine relative activity of the tested peptides. All procedures were carried out according to the literature recommendations (Choma and Grzelak 2011). Specifically, all tests involved different times of incubation, evaporation, temperature, concentrations of initial inoculums and various concentrations of the tested compounds in order to choose the most suitable and reproducible method. Additionally, different types of TLC plates and separation buffers were investigated. In many cases they were not suitable to perform microbiological tests. Moreover, to visualize the results a resazurin solution (4 mg/mL, 0.1 % Triton X-100) was used. According to the literature, visualization of antimicrobial activity involves the use of aqueous solutions of tetrazolium salts and incubation in humid chambers as well (Choma and Grzelak 2011; Choma et al. 2012; Horvath et al. 2002; Kalaivani and Vidhya 2014). In our study enhanced humidity only extended the incubation time. TLC-DB without development of the mobile phase revealed compounds with antimicrobial activity which was proved by MIC results obtained by broth microdilution method. Spots of six out of the nine tested antimicrobial peptides namely, CAMEL, Citropin 1.1, Pal-KK-NH_2_, Pal-KGK-NH_2_, dimeric lipopeptide, Laur-CKK-NH_2_ and Mir-KGK-NH_2_ did not reduce resazurin. Inactive peptides did not inhibit bacterial and fungal growth on TLC plates. However, during separation of peptide ICHHCI-OH, a white inhibition zone was spotted for all microbial strains. For plates without separation the soft white inhibition zone was also observed. This result deserved the explanation of possible reasons. Consequently it was concluded that the use of resazurin was inadequate in case of antibiotic peptides containing cysteine residues with free thiol groups, as they reduce resazurin to colourless hydroresorufin. That process is utilized, for example, in reducing media for cultivation of anaerobic organisms (Fukushima et al. 2002, 2003). Therefore, peptides with reducing properties vitiate the results. The spots might have been interpreted as growth-inhibition zones. This problem does not concern peptides containing cysteine residues linked by a disulfide bond blocking reactive thiol groups. It was confirmed with the dimeric lipopeptide, Laur-CKK-NH_2_. Also for this peptide, two inhibition zones were visualized during separation. Consequently, it might have been concluded that the crude solution was represented by two antimicrobial fractions. In the study analogues of lipopeptides were also investigated. One of them, Mir-KGK-NH_2_, displayed the same antimicrobial activity against Gram-positive strains of bacteria as Pal-KGK-NH_2_ and lower against Gram-negative strains. Therefore, the difference in inhibition zones was not observed, which suggest that our method is rather qualitative than quantitative. Another analogue, Pal-K(TFA)K(TFA)-NH_2_ was also tested. In this case, the presence of TFA protecting groups on lysine dramatically changed the activity of compound which was confirmed by TLC-DB and by broth microdilution assay.

All obtained results for both methods: with and without separation were comparable for all microorganisms tested. The inhibition zones were always observed for active compounds, also in case of peptides that exhibited very high inhibitory concentration (for example MIC values of all tested compounds against *C. albicans* were above 64 µg/mL and were still correctly interpreted, Fig. 1c). This results indicate that compounds with any antimicrobial activity can be easily displayed.

According to the results, our study would have an application, especially for screening a large number of compounds. The TLC-DB method is suitable for both Gram-positive and Gram- negative bacteria and fungi as well. So far, the results obtained with peptides containing cysteine residues containing free thiol groups should be carefully interpreted. In this case, the method offers the possibility of screening active peptides rapidly and efficiently.
